# Ampullary carcinoma detected incidentally during ERCP: a case report and literature review

**DOI:** 10.3389/fonc.2026.1840198

**Published:** 2026-05-20

**Authors:** Heyue Zhang, Wei Wu, Jin Xu

**Affiliations:** Department of First General Surgery, The Sixth People’s Hospital of Shenyang, Shenyang, Liaoning, China

**Keywords:** ampulla of Vater, ampullary carcinoma, case report, double duct sign, endoscopic retrograde cholangiopancreatography

## Abstract

Early ampullary tumors are challenging to diagnose due to their complex surrounding structures and deep anatomical location. We report a rare case in which enhanced computed tomography (CT), magnetic resonance imaging (MRI), magnetic resonance cholangiopancreatography (MRCP) and endoscopic ultrasound (EUS) all failed to detect the lesion, with the diagnosis ultimately established through endoscopic retrograde cholangiopancreatography (ERCP). The patient subsequently underwent timely surgical resection. This case highlights the irreplaceable value of ERCP in detecting early and occult ampullary tumors.

## Introduction

The ampulla of Vater is located at the confluence of the common bile duct and the pancreatic duct (1). Most patients with ampullary carcinoma present with obstructive jaundice, prompting detection of the mass. However, early tumors often manifest with atypical symptoms, rendering them easily overlooked and resulting in delayed diagnosis (2). Furthermore, the complex surrounding anatomy and deep location of the ampulla pose significant challenges in accurately determining the tumor’s location and nature. Early diagnosis and timely treatment are crucial prognostic factors for patients with ampullary carcinoma.

In previous studies, ampullary tumors diagnosed exclusively by ERCP without detection by other imaging modalities have been extremely rare. This article describes the clinical manifestations, diagnostic workup, and therapeutic management of such a case. Furthermore, we conducted a systematic review of similar cases reported in the literature to provide novel insights into early diagnosis and treatment strategies.

## Case description

A 60-year old male presented with a 3 day history of upper abdominal distension and pain. The pain was postprandially aggravated and accompanied by low grade fever. Notably, he had experienced approximately 4 kg of unintentional weight loss over the preceding month. Laboratory investigations revealed the following abnormalities: white blood cell count 11.34 × 10^9^/L; C-reactive protein 30.3 mg/L; total bilirubin 26.5 μmol/L; alanine transaminase 64.9 U/L; gamma-glutamyl transferase 329 U/L; serum amylase 400 U/L; serum lipase 939.3 U/L; and urinary amylase 1000 U/L.

**Figure 1:**
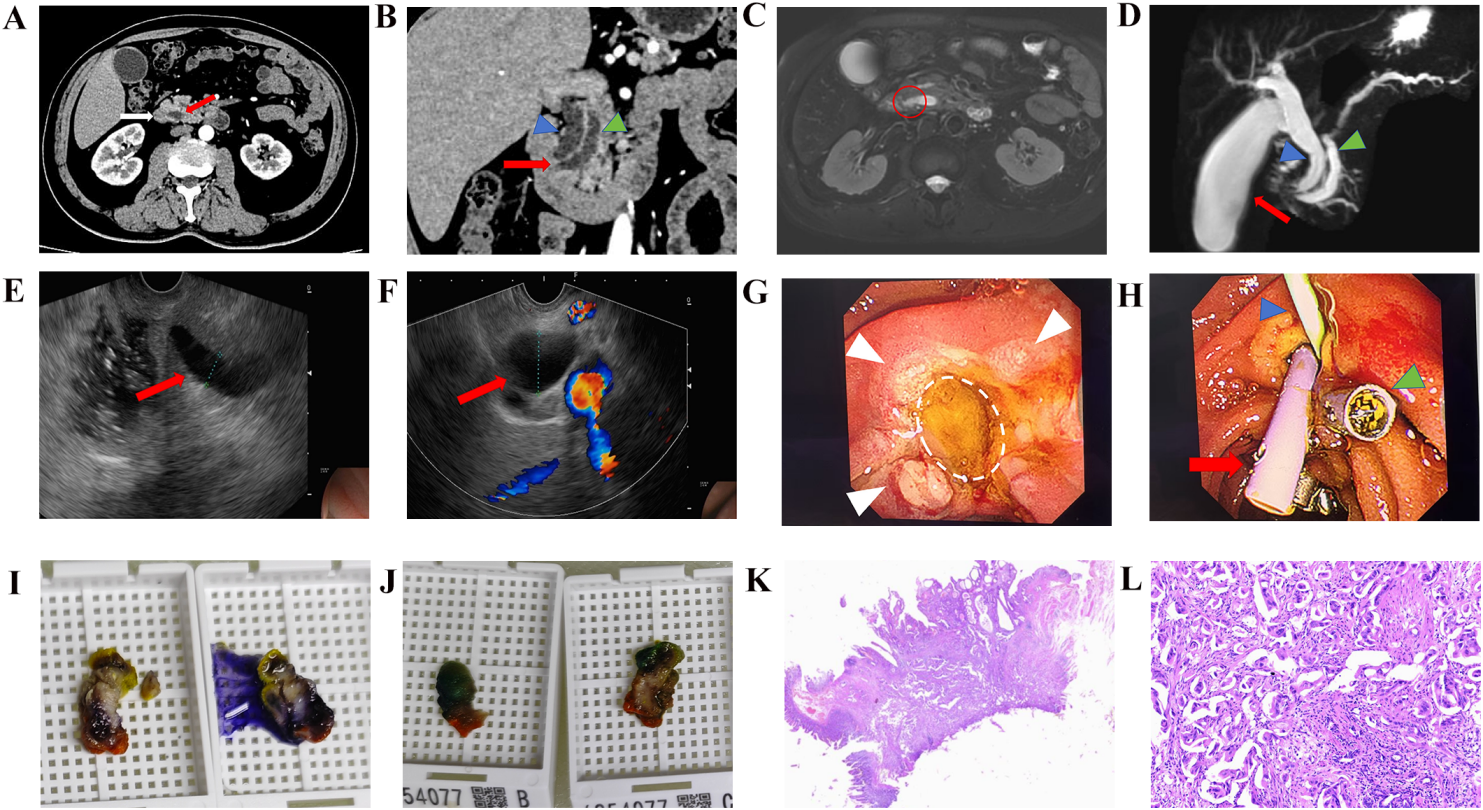
Imaging studies, endoscopic findings, and histopathological examination of the patient. **(A)** Contrast-enhanced CT showing no mass at the ampulla of Vater. The red arrow indicates the confluence of the bile and pancreatic ducts; the white arrow indicates the duodenum and major duodenal papilla. **(B)** Coronal view of CT demonstrating the DDS. The blue mark indicates the dilated common bile duct; the green mark indicates the dilated pancreatic duct; the red arrow indicates the junction of the two ducts. **(C)** T2-weighted sequence of MRI. The red circular area corresponds to the ampulla, with no occupying lesion identified. **(D)** MRCP revealing the DDS.(pointed by the blue and green marks) and distended gallbladder(indicated by the red arrow). **(E)** EUS of the common bile duct (indicated by the red arrow), with the dilated segment measuring approximately 1.6 cm in maximal diameter. **(F)** EUS of the dilated pancreatic duct (indicated by the red arrow). **(G)** EST of the major duodenal papilla. The white marks indicate the remaining tumor tissue. The white circle indicates the cut surface after ampullary biopsy. **(H)** Placement of pancreatic duct stent (indicated by the blue mark) and bile duct stent (indicated by the red arrow); closure of the papillectomy defect with endoscopic hemoclips(indicated by the green mark). **(I, J)** Gross specimens of the resected ampullary tumor and adjacent tissues. **(K, L)** Histopathological examination (hematoxylin-eosin staining) confirming intestinal-type papillary adenocarcinoma.

The patient was diagnosed with acute pancreatitis and admitted for anti-inflammatory therapy and pancreatic enzyme inhibition. Concomitant imaging studies were performed (**Figures 1A–F**), all demonstrating the double-duct sign (DDS) without evidence of space-occupying lesions. Although the patient remained anicteric and symptomatically improved with conservative management, rendering immediate further intervention unnecessary, close follow up was advised. However, given that the DDS is a significant indicator of malignancy in the pancreatobiliary system, albeit occasionally observed in benign or inflammatory conditions, we elected to proceed with ERCP to exclude an occult neoplasm. Endoscopic examination revealed no abnormal protrusions on the duodenal papillary surface and no periampullary diverticulum. Following Endoscopic Sphincterotomy (EST), a lesion was visualized at the confluence of the common bile duct and pancreatic duct within the ampulla(**Figure 1G**). Ampullary biopsy were obtained for histopathological analysis (**Figures 1I, J**), and pancreaticobiliary stents were placed (**Figure 1H**). Histopathological examination confirmed intestinal-type ampullary adenocarcinoma (**Figures 1K, L**). The patient subsequently underwent radical pancreaticoduodenectomy (Whipple procedure). The surgery went smoothly. Postoperatively, he developed transient hypoproteinemia and blood glucose fluctuations, both of which resolved with conservative management. No other complications were encountered, and the postoperative hospital stay was 17 days. Chemotherapy with the FOLFOX regimen was initiated 46 days after surgery; however, after completing four cycles, the patient discontinued treatment due to adverse effects, including profound gastrointestinal toxicity and malnutrition. To date, at 8 months of follow-up, there has been no evidence of tumor recurrence.

## Discussion

The ampulla of Vater is a short, funnel shaped channel formed by the confluence of the common bile duct and the main pancreatic duct within the duodenal wall, opening onto the medial aspect of the descending duodenum via the major duodenal papilla. Ampullary carcinoma may arises from the center of the ampulla, its periphery, or may completely involve the ampullary complex. These neoplasms are relatively uncommon, accounting for only 0.6%–0.8% of gastrointestinal malignancies (2). Initial clinical manifestations are often nonspecific, including abdominal distension, pain, jaundice, and unintentional weight loss. These symptoms that are frequently overlooked, resulting in delayed diagnosis and treatment (3). Early detection and timely intervention remain the only means of improving prognosis.

Currently, contrast-enhanced CT,MRI and MRCP represent the most widely utilized and preferred imaging modalities for evaluating ampullary region tumors. However, the diagnostic accuracy of both modalities is approximately 70% (2). This limitation stems from the deep anatomical location of the ampulla, its narrow and conical configuration, and consequently poor spatial resolution. Additionally, the presence of intraluminal gas and fluid within the ampulla may create artifacts that compromise diagnostic accuracy, particularly for centrally located lesions (4). EUS substantially improves the detection of early ampullary carcinoma. A multicenter study demonstrated that EUS achieves a sensitivity of 0.89 and specificity of 0.87 for T1-stage ampullary neoplasms (5). The European Society of Gastrointestinal Endoscopy recommends EUS with fine-needle aspiration (EUS-FNA) as the preferred diagnostic modality when ampullary malignancy is suspected (6). Although ERCP remains the sole nonsurgical technique enabling direct visualization of the bile duct, pancreatic duct, and major duodenal papilla, its invasive nature carries significant risks of serious complications, including pancreatitis, hemorrhage, and perforation. Furthermore, tissue architectural alterations induced by sphincterotomy and biliary or pancreatic stent placement may reduce the accuracy of subsequent histopathological evaluation by up to 25% (7). Consequently, the diagnostic role of ERCP remains highly controversial.

For patients exhibiting the DDS without concurrent clinical jaundice, current guidelines do not specify a standardized diagnostic pathway. Some investigators advocate that EUS-FNA offers sufficient diagnostic accuracy, thereby avoiding the additional procedural risks inherent to ERCP (8). When EUS-FNA yields negative results, a strategy of symptomatic management combined with structured follow-up surveillance may be adopted (9). However, an opposing viewpoint maintains that ERCP should still be pursued in patients with adequate physiological reserve, notwithstanding the associated healthcare resource utilization, to ensure definitive exclusion of malignant disease (10).

Retrospective analysis of this case reveals that ERCP, employed as a supplemental diagnostic modality, successfully identified an ampullary carcinoma that remained undetected by the combined imaging battery of contrast-enhanced CT, MRI,MRCP, and EUS. This finding underscores the inherent limitations of conventional examinations in detecting early ampullary neoplasms. The underlying mechanism likely relates to tumor concealment within the papillary mucosal folds or complete intraluminal growth within the pancreatobiliary channel without surface protrusion, scenarios that create significant blind spots for cross sectional and endoscopic imaging modalities. Furthermore, this case demonstrates that the DDS may manifest at a very early stage of ampullary tumorigenesis, preceding morphological alterations of the major duodenal papilla, and thus constitutes a critical indicator that warrants immediate investigation rather than observational follow up. EST combined with ampullary biopsy can directly access and remove the tissue, which theoretically can increase the positive rate of biopsy. Direct intraluminal visualization via ERCP is imperative to preclude diagnostic delay. Given the cumulative procedural burden and potential morbidity associated with sequential EUS and ERCP examinations, some investigators have proposed a hybrid diagnostic approach the “endoscopic ultrasonography-retrograde cholangiopancreatography” concept, wherein both invasive procedures are completed in a single endoscopic session (11). Although not yet widely adopted, this integrated strategy holds considerable promise for future clinical application.

Furthermore, we conducted a search in PubMed from 2000 to the present, retrieving literature reports where only the DDS was observed through CT, US, EUS, MRI, MRCP, etc (12–17). And where a papillary tumor of the ampulla was discovered through ERCP (**Table 1**). Among the above 6 patients, 3 cases were diagnosed through ERCP biopsy, 1 case was confirmed by re-examination of EUS, and 2 cases were diagnosed through surgery. There were 4 cases of ampullary adenocarcinoma, 1 case of ampullary adenoma, and 1 case of somatostatinoma. In Case 1, due to negative ERCP biopsy results, regular follow-up was conducted. The interval from the initial visit to the final biopsy was 18 months (12). Eventually, the surgical opportunity was lost. In Case 4, an extremely early lesion in the ampulla was detected during the first ERCP, and the pathology was negative (15). A decision was made to undergo follow-up. Eventually, a biopsy was obtained through bile duct brushing, and the postoperative pathology indicated that the ampulla cancer had invaded the lower segment of the common bile duct. While in Case 2 and Case 6, although no cancer was found in the biopsy, immediate surgery was chosen, resulting in timely diagnosis and treatment (13, 17). It can be seen that ERCP, as a supplementary examination method, can increase the detection rate, but it should not be regarded as the gold standard or the endpoint for diagnosis. When the double-tube sign appears and ERCP reveals a lesion in the ampulla but the biopsy is negative, surgery should be the final treatment method. Another study shows that the diagnostic positive rate of ERCP is only 50%, that is, diagnosing non-malignant lesions in the extracted biopsy samples does not rule out the possibility of ampullary cancer (18).

**Table 1:** Literature review of cases.

Case	Age /sex	Clinical present	Imaging results	Therapeutic method	Pathological diagnosis
1 (12)	74/M	Progressive jaundice weight loss	US(-), MRCP(-),1^st^ of EUS (-) ERCP:ampullary mass(+), biopsied(-) Repeat EUS:biopsied(+)	Chemotherapy	ampullary adenocarcinoma
2 (13)	78/F	nausea, weight loss, jaundice	MRCP(-); EUS(-);CT(-) ERCP:ampullary mass (+), biopsied(-)	pylorus-preserving pancreaticoduodenectomy	ampullary adenocarcinoma
3 (14)	–	–	MRI(-); CT(-) ERCP:ampullary mass (+), biopsied(+)	Endoscopic resection	ampullary adenoma
4 (15)	79/F	chillness; nausea; cholangitis	CT(-); 1^st^ of ERCP:ampullary mass (+), biopsied(-) Final ERCP:biopsied (+)	Whipple’s procedure	papillary carcinoma of the duodenum
5 (16)	74/F	epigastric pain	CT(-);MRI(-);1^st^ of ERCP:ampullary mass (-), 3^rd^ of ERCP:ampullary mass (+), biopsied(+)	Whipple’s procedure	ampullary adenocarcinoma
6 (17)	43/M	(-)	CT(-);MRI(-);MRCP(-) ERCP:ampullary mass (+), biopsied(-)	Whipple’s procedure	Somatostatinoma

M, male; F, female; 1^st^, first time; 3^rd^, third time; (+),positive result; (-), negative result.

## Conclusion

ERCP holds significant value in the diagnosis of early and occult ampullary neoplasms, and should be considered as a complementary approach when endoscopic ultrasonography yields negative findings. However, when an ampullary lesion is identified but biopsy yields negative results, timely surgical intervention is preferable to observational follow up.

## Data Availability

The datasets presented in this study can be found in online repositories. The names of the repository/repositories and accession number(s) can be found in the article/supplementary material.
